# Among‐years rain variation is associated with flower size, but not with signal patch size in *Iris petrana*

**DOI:** 10.1002/ecy.3839

**Published:** 2022-09-25

**Authors:** Sissi Lozada‐Gobilard, Allyson Motter, Yuval Sapir

**Affiliations:** ^1^ The Botanical Garden, School of Plant Sciences and Food Security Faculty of Life Sciences, Tel Aviv University Tel Aviv Israel; ^2^ Department of Biology University of Virginia Charlottesville Virginia USA

**Keywords:** floral signal, flower size variation, flower traits, plasticity, pollination, rainfall, Royal Irises, selection

The vast diversity of flower shapes, colors, and sizes is considered a classic example of coevolution and adaptation. Plants use flowers to attract pollinators and offer them rewards, such as nectar, pollen or resins, in exchange for pollen transfer to flowers of other conspecific plants (Faegri & van der Pijl, 1979). Size of flowers is a visual cue that facilitates their detection from a distance (Schiestl & Johnson, 2013). Flower size is associated with the amount of the reward, in which larger flowers are usually a signal for higher energetic and nutritive rewards than smaller ones (Ortiz et al., 2021). Indeed, many studies have shown that pollinators prefer larger flowers, resulting in pollinator‐mediated directional selection on flower size (Teixido et al., 2016).

Although pollinators select for larger flowers, large flowers are costly to produce and maintain, and smaller flowers may be favored under certain environmental conditions, exerting environmentally mediated selection on flower size (Caruso et al., 2018; Roddy, 2019; Teixido et al., 2016). Flower development and growth highly depend on water availability causing a reduction in flower size in drought conditions (Gallagher & Campbell, 2017). Smaller flowers fit better to drought and other abiotic stress, and therefore, in arid environments the selection by pollinators and abiotic factors is likely to be conflicting.

Both drought‐ and pollinator‐mediated selection are acting on flower size, but due to modularity of flower traits, other floral cues could respond differently to these selection agents. Berg's hypothesis suggests that different parts of the flower experience different selection regimes, resulting in contrasting patterns of variation and plasticity (Berg, 1960). This may be reflected in intrafloral decoupling of plastic response to environmental perturbations. Although flower size is a trait that shows extensive plasticity within individuals or species, unique signal traits that show relatively few transitions within and among plant lineages (e.g., floral color patterns) are expected to show little or no plasticity (Givnish, 2002). Here we report on this decoupling between floral size and flower color signal in *Iris petrana* Dinsm., a desert species of the Royal Irises.

Royal Irises (*Iris* section *Oncocyclus*) are herbaceous geophytes, consisting of 65 species endemic to the Middle East that serve as model system for speciation, pollination and ecology (Roguz et al., 2020; Sapir et al., 2002). They produce one large flower per stem and are fully self‐incompatible, therefore depending on pollinators for successful reproduction (Sapir et al., 2005). Their major pollinators are male solitary bees of the genus *Eucera* that use the flowers as night shelters, transporting pollen while looking for suitable flowers at sunset (Sapir et al., 2005). The dark color of the flowers may increase the temperature of the pollination tunnel in the morning, where bees shelter, offering a passive heat reward that helps them emerge to fly in the morning (Monty et al., 2006; Sapir et al., 2006).

Apart from their exceptional flower size and color, all species of the Royal Irises share a unique phenotypic feature: a dark circle signal in the middle of the fall lower tepals (Figure 1a), from this point forward referred to as the “black patch.” Little information is known about the role and evolutionary significance of the black patch and its relationship with flower size and whether it is under selection. Previous studies have suggested that the large size and dark color of Royal Irises are under pollinator‐mediated selection to mimic dark shelters, but no clear function of the black patch in attracting pollinators has been identified (Vereecken et al., 2013). The black patch might be involved in heating the tunnel where the bees shelter, absorbing the morning sun radiation and transferring the heat to the pollination tunnel at sunrise (Lozada‐Gobilard et al., in preparation).

**FIGURE 1:**
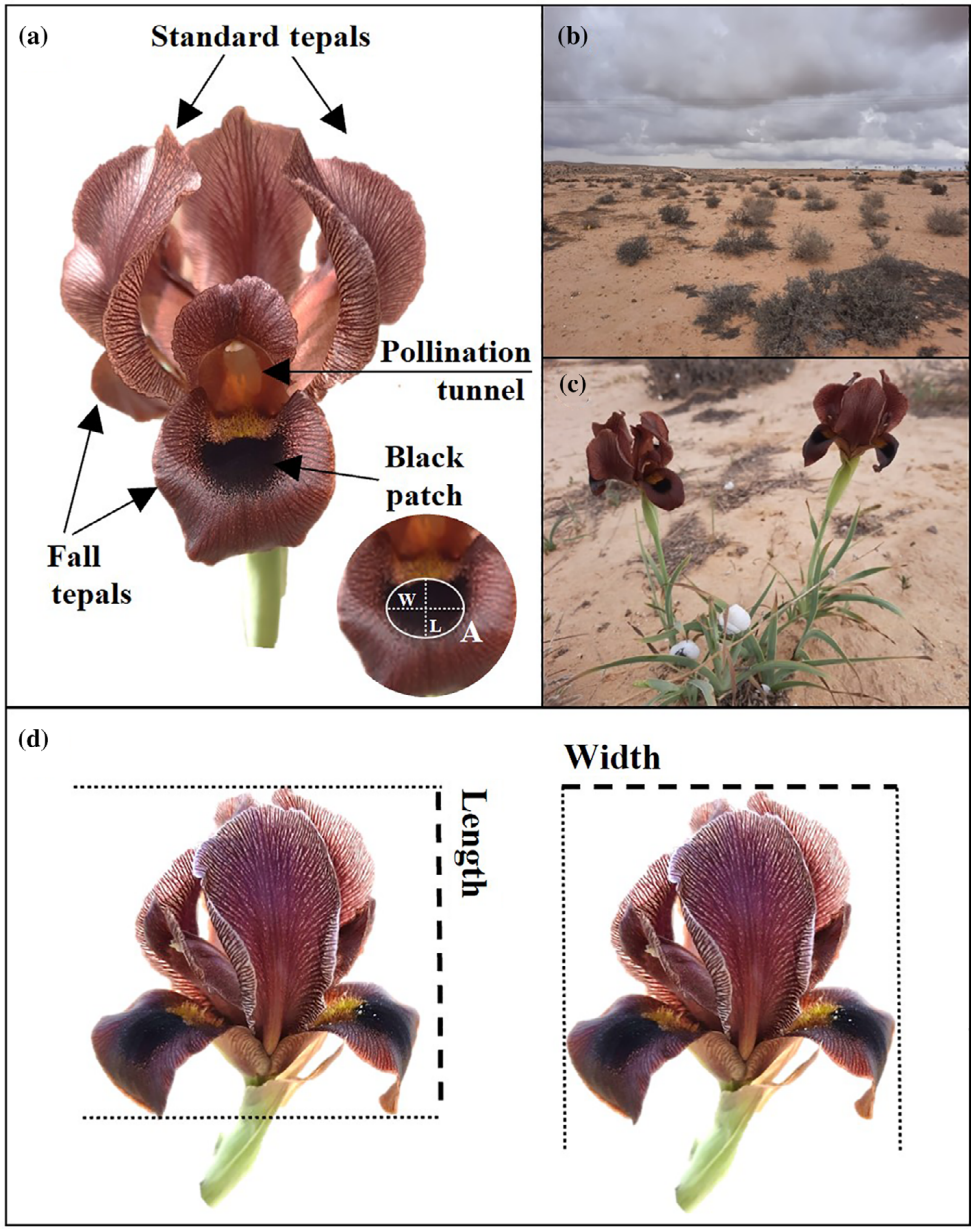
Flower structure of *Iris petrana* (a), study site Yeruham Nature Reserve (b), one individual plant (genet) with five ramets and two flowers (c) and flower size measurements (d). The pollination tunnel is formed by the petaloid style with a stigmatic surface at the tip, one anther is inside and a landing platform, “beard” hairs (in yellow) and black patch at the center of the fall tepal. Each flower has three pollination tunnels. Projection size was calculated by multiplying length × width (d). Black patch area was calculated multiplying length and width of the black patch measured in one of the three fall tepals, or as direct area using ImageJ software (a).

Although flower size in irises has been investigated as a signal to pollinators (Lavi & Sapir, 2015), less attention has been given to the size of the black patch. Its size only varies slightly among populations and species (Sapir et al., 2002), but nothing is known about its role as visual cue to pollinators, its plastic response to environmental variation or the selection forces acting on it. Flower size in the Royal Irises was shown to decrease with increased aridity along the north–south precipitation gradient in Israel (Arafeh et al., 2002; Sapir et al., 2002). *I. petrana* grows in desert habitats, with a mean annual precipitation of 110 mm, but with a high variation among years (Appendix S1: Figure S1). These changes in annual rain among years might affect the size of the flowers and the size of the black patch, potentially affecting plant–pollinator interactions and plant fitness. In this study, we tested whether flower size and black patch size are associated with changes in annual rain using historical data from the turn of the millennium (1998/1999; Sapir et al., 2002), and recent data measured in the field (2017–2021).

We studied a population of *Iris petrana* located in the Yeruham *Iris* Nature Reserve south of Israel (31°01′14.46 N, 34°58′21.4 E, altitude 557 m) in the northern part of the Negev desert (Figure 1b). This population consists of hundreds of different genets (genetical identical individuals) distributed in well defined patches (Figure 1c) in an area of ~480 km^2^. Each individual produces on average 2 ± 1 flowers. This site has 110 mm of mean annual rain, all during the winter (October to March) and a mean annual temperature of 18.3°C. The highest rainfall values occur between December and February (Appendix S1: Figure S2), with very little or no rain from June to August. *I. petrana* is a rhizomatous perennial herb, able to grow vegetatively. Its flowering season is short, from the second week of March to middle of April, and each single flower remains open from 3 to 5 days. Fruits and seeds ripen 2–3 weeks after the flower wilts, if the flower was pollinated.

Flower size was measured in the field using calipers, during the flowering season (March–April in 1998, 1999, 2017, 2019, 2020, and 2021). We measured flower length and flower width of the entire flowers and the black patch located in the fall tepals. Flower projection size was estimated by multiplying the length by the width of the entire flower (Figure 1d). Black patch area was similarly calculated by multiplying length by width (Figure 1a), and relative black patch area dividing the black patch area by the total area of the flower. In the flowering season of 2017, 2020, and 2021, we estimated the black patch area from digital photographs taken in the field, using ImageJ software (Schneider et al., 2012). Data collected in 1998–1999 correspond to the beginning of the Iris research by Y. Sapir (Sapir et al., 2002). The later measurements were part of the yearly monitoring of *I. petrana* populations conducted by the Sapir research group at Tel Aviv University. New individuals were measured every time and therefore sample sizes varied among years (Appendix S1: Table S1).

Daily rain data were downloaded from the Israeli Meteorological Service (IMS) online (https://ims.data.gov.il/en). Annual rain was calculated as a mean of the four nearest meteorological stations to Yeruham Iris Reserve (Appendix S1: Table S1). These are Beer Sheva (40 km north), Sede Boker (23 km southwest), Zomet Hanegev (12.5 km east) and Mizpe Ramon (40 km southwest). The rainy season in Yeruham spans from September to May of the next year, therefore the accumulated rain from these months was used to determine annual rain for each season. To test whether rain varies among years, we performed an ANOVA test combined with Tukey post hoc tests for pairwise comparisons. To test whether there is an association between annual rain and flower size and black patch (total and relative size), we performed linear models separately by variable. In addition, to test for the effect of rainfall on the black patch area, correcting for flower size, we performed an ANCOVA test in which patch size depends on rainfall, including flower size as a co‐variate. Variables were *sqrt* transformed to achieve normality. All statistics were performed in R version 4.1.2 (R Core Team, 2019).

Rain was highly variable among years (*F*_5,18_ = 6.3, *p* < 0.01), and ranged between 22.1 (season 2020–2021) to 257.4 mm (season 1999–2000; Figure 2a). Flower size increased significantly with mean annual rainfall (*F*_1,945_ = 632.7, *p* < 0.001; Figure 2b), but did not change with total black patch area (*F*_1,744_ = 0.395, *p* = 0.529; Figure 2c). Consequently, the percentage of patch area declined with mean annual rainfall (*F*_1,744_ = 190.83, *p* < 0.001; Figure 2d). According to an ANCOVA test, the black patch area was significantly associated with both flower size (*F*_1,742_ = 68.7, *p* < 0.001), and annual rain (*F*_1,742_ = 32.8, *p* < 0.001), as well as their interaction (*F*_1,742_ = 18.4, *p* < 0.001; Appendix S1: Table S2). Nonetheless, effect size of flower size was an order of magnitude higher than the effect size of rain (0.038 and 0.004 cm^2^/mm, respectively; Appendix S1: Table S2).

**FIGURE 2:**
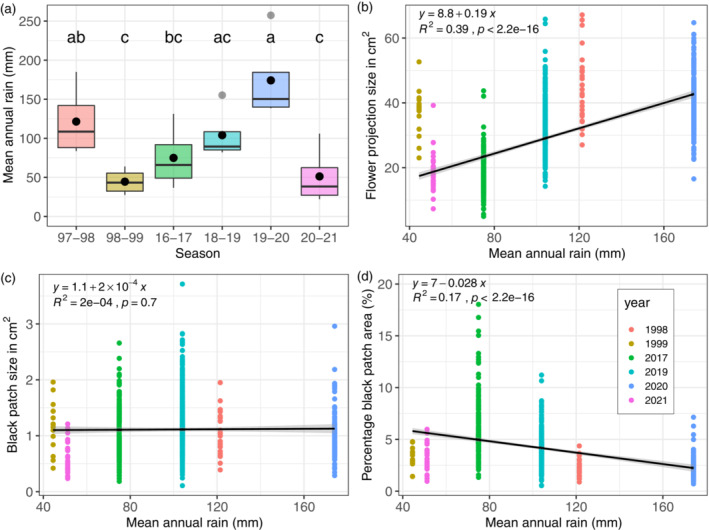
Annual rain, variation in time, and its association with flower size and black patch. Annual rain varied among years (a), and showed a positive association with flower projection size (b); whereas black patch area remained constant (c). Annual rain negatively associated with percentage black patch area also varied in time inversely related to flower size (d). Black points in the middle of the boxplots represent mean values.

These results suggest that variation in annual rain drives flower size changes in *I. petrana*. This is expected in desert populations, in which water availability is the most vital element, and growth of plants highly depends on it. Increased variation in seasonal rain and abrupt decreases such as the one observed in 2021, as predicted with current and future climate changes, can increase plants vulnerability with possible negative fitness consequences. Drought stress can cause flower size reduction, affecting plant–pollinator interactions, increasing pollen limitation, and therefore decreasing fitness (Gallagher & Campbell, 2017). In addition to morphological changes, extreme climate perturbation may lead to a reduction in display size, and less flower production could eventually lead to less reproduction output and overall reduction in population plant fitness. Indeed, in 2021 the extreme drought caused a decrease in number of flowers: only ~60 flowers developed in that year, while normally tens of thousands of flowers bloom. Consequently, only one fruit was found in the whole population (S. Lozada‐Gobilard, pers. observ.).

High variation of flower size in relation to rain is likely to be a plastic response, rather than selection (Givnish, 2002). A plastic response in flower size is likely to be due to the perennial lifeform of these plants, whose rhizomes can stay in the soil for many years (up to 60) and resprout new leaflets and flowers every season. More interestingly, despite the high variation in flower size, the absolute area of the black patch was constant over the years (Figure 2c). This suggests that the black patch size and flower size might be decoupled in their response to rain variation. If this is true, we hypothesize that the size of the black patch might be an important visual cue to attract pollinators or be involved in flower temperature and heat reward (or both; Lozada‐Gobilard et al., in preparation). Therefore, even under stressful conditions (i.e., low water availability), the plant will invest resources to keep the area of the black patch constant to assure pollinators visits. However, whether the black patch size indeed attracts more pollinators and enhances fitness remains unclear.

Our results show that high flower size variation could be explained by fluctuations in rain over the seasons. However, the black patch area remained constant and did not depend on annual rainfall. These results suggest that patch size is important, and that the plant is investing resources to keep the area of the black patch constant. This work raises questions about the selection forces acting on these floral traits. These results might be an indication that flower size and the black patch size are under different selection regimes. Although flower size might be under a putatively balancing selection of both pollinators and climate, the black patch might be putatively selected by pollinators only. More studies are needed to investigate the evolutionary and selection drivers of these particular flower traits on this and other *Oncocyclus* species.

## AUTHOR CONTRIBUTIONS

Sissi Lozada‐Gobilard and Yuval Sapir designed the study, Yuval Sapir and Sissi Lozada‐Gobilard collected some of the data. Allyson Motter and Sissi Lozada‐Gobilard performed the data curation and statistics. Sissi Lozada‐Gobilard led the writing of the manuscript. All coauthors contributed significantly with helpful discussions and worked together on the final version of the manuscript.

## CONFLICT OF INTEREST

The authors have no conflicts of interest to declare.

## Supporting information


Appendix S1

## Data Availability

Data and R script code (Lozada Gobilard et al., 2022) are available on Figshare at https://doi.org/10.6084/m9.figshare.19493654.v2. Raw data of rainfall were downloaded from the Israeli Meteorological Service (IMS) (https://ims.data.gov.il/) by following these steps: (1) Meteorological Data Archive, (2) Daily rain, (3) From: 1 January 1979 Until: 31 December 2021, (4) Beersheva, Sde Boker, Mitzpe Ramon, Negev, (5) By stations, (6) Generate report.

## References

[ecy3839-bib-0001] Arafeh, R. M. H. , Y. Sapir , A. Shmida , N. Iraki , O. Fragman , and H. P. Comes . 2002. “Patterns of Genetic and Phenotypic Variation in *Iris haynei* and *I. atrofusca* (Iris Sect. Oncocyclus = the Royal Irises) along an Ecogeographical Gradient in Israel and the West Bank.” Molecular Ecology 11: 39–53. 11903903 10.1046/j.0962-1083.2001.01417.x

[ecy3839-bib-0002] Berg, R. L. 1960. “The Ecological Significance of Correlation Pleiades.” Evolution 14: 171–80.

[ecy3839-bib-0003] Caruso, C. M. , K. E. Eisen , R. A. Martin , and N. Sletvold . 2018. “A Meta‐Analysis of the Agents of Selection on Floral Traits.” Evolution 73: 4–14. 30411337 10.1111/evo.13639

[ecy3839-bib-0004] Faegri, K. , and L. van der Pijl . 1979. “Applied Pollination Ecology.” In The Principles of Pollination Ecology. Oxford, UK: Pergamno Press.

[ecy3839-bib-0005] Gallagher, M. K. , and D. R. Campbell . 2017. “Shifts in Water Availability Mediate Plant–Pollinator Interactions.” New Phytologist 215: 792–802. 28517023 10.1111/nph.14602

[ecy3839-bib-0006] Givnish, T. J. 2002. “Ecological Constraints on the Evolution of Plasticity in Plants.” Evolutionary Ecology 16: 213–42.

[ecy3839-bib-0007] Lavi, R. , and Y. Sapir . 2015. “Are Pollinators the Agents of Selection for the Extreme Large Size and Dark Color in Oncocyclus Irises?” New Phytologist 205: 369–77. 25157604 10.1111/nph.12982

[ecy3839-bib-0008] Lozada Gobilard, S. , A. Motter , and Y. Sapir . 2022. “Relation between Annual Precipitation and Flower Traits of Iris Petrana.” Figshare. 10.6084/m9.figshare.19493654.v2.

[ecy3839-bib-0009] Monty, A. , L. Saad , and G. Mahy . 2006. “Bimodal Pollination System in Rare Endemic Oncocyclus Irises (Iridaceae) of Lebanon.” Canadian Journal of Botany 84: 1327–38.

[ecy3839-bib-0010] Ortiz, P. L. , P. Fernández‐Díaz , D. Pareja , M. Escudero , and M. Arista . 2021. “Do Visual Traits Honestly Signal Floral Rewards at Community Level?” Functional Ecology 35: 369–83.

[ecy3839-bib-0011] R Core Team . 2019. R: A Language and Environment for Statistical Computing. Vienna: R Foundation for Statistical Computing.

[ecy3839-bib-0012] Roddy, A. B. 2019. “Energy Balance Implications of Floral Traits Involved in Pollinator Attraction and Water Balance.” International Journal of Plant Sciences 180: 944–53.

[ecy3839-bib-0013] Roguz, K. , M. K. Gallagher , E. Senden , Y. Bar‐Lev , M. Lebel , R. Heliczer , and Y. Sapir . 2020. “All the Colors of the Rainbow: Diversification of Flower Color and Intraspecific Color Variation in the Genus *Iris* .” Frontiers in Plant Science 11: 1–16. 33154761 10.3389/fpls.2020.569811 PMC7588356

[ecy3839-bib-0014] Sapir, Y. , A. Shmida , O. Fragman , and H. P. Comes . 2002. “Morphological Variation of the Oncocyclus Irises (*Iris*: Iridaceae) in the Southern Levant.” Botanical Journal of the Linnean Society 139: 369–82.

[ecy3839-bib-0015] Sapir, Y. , A. Shmida , and G. Ne'eman . 2005. “Pollination of Oncocyclus Irises (Iris: Iridaceae) by Night‐Sheltering Male Bees.” Plant Biology 7: 417–24. 16025415 10.1055/s-2005-837709

[ecy3839-bib-0016] Sapir, Y. , A. Shmida , and G. Ne'eman . 2006. “Morning Floral Heat as a Reward to the Pollinators of the Oncocyclus Irises.” Oecologia 147: 53–9. 16205954 10.1007/s00442-005-0246-6

[ecy3839-bib-0017] Schiestl, F. P. , and S. D. Johnson . 2013. “Pollinator‐Mediated Evolution of Floral Signals.” Trends in Ecology & Evolution 28: 307–15. 23480953 10.1016/j.tree.2013.01.019

[ecy3839-bib-0020] Schneider, C. A., W. S. Rasband, and K. W. Eliceiri. 2012. “NIH Image to ImageJ: 25 Years of Image Analysis.” Nature Methods 9: 671–5. 10.1038/nmeth.2089 PMC5554542 22930834

[ecy3839-bib-0018] Teixido, A. L. , M. Barrio , and F. Valladares . 2016. “Size Matters: Understanding the Conflict Faced by Large Flowers in Mediterranean Environments.” Botanical Review 82: 204–28.

[ecy3839-bib-0019] Vereecken, N. J. , A. Dorchin , A. Dafni , S. Hötling , S. Schulz , and S. Watts . 2013. “A Pollinators' Eye View of a Shelter Mimicry System.” Annals of Botany 111: 1155–65. 23599249 10.1093/aob/mct081 PMC3662522

