# Supplementary material for: Among‐years rain variation is associated with flower size, but not with signal patch size in Iris petrana
Source: Ecology. 2022 Sep 25;104(1):e3839. doi: 10.1002/ecy.3839 (PMC10078192; doi:10.1002/ecy.3839)
Supplement: Supplementary file 1 — Appendix S1 [file ECY-104-0-s001.pdf]

ECOLOGY

Among-years rain variation is associated with flower size, but not with signal patch size in *Iris petrana*

Sissi Lozada-Gobilard, Allyson Motter, and Yuval Sapir

Appendix S1

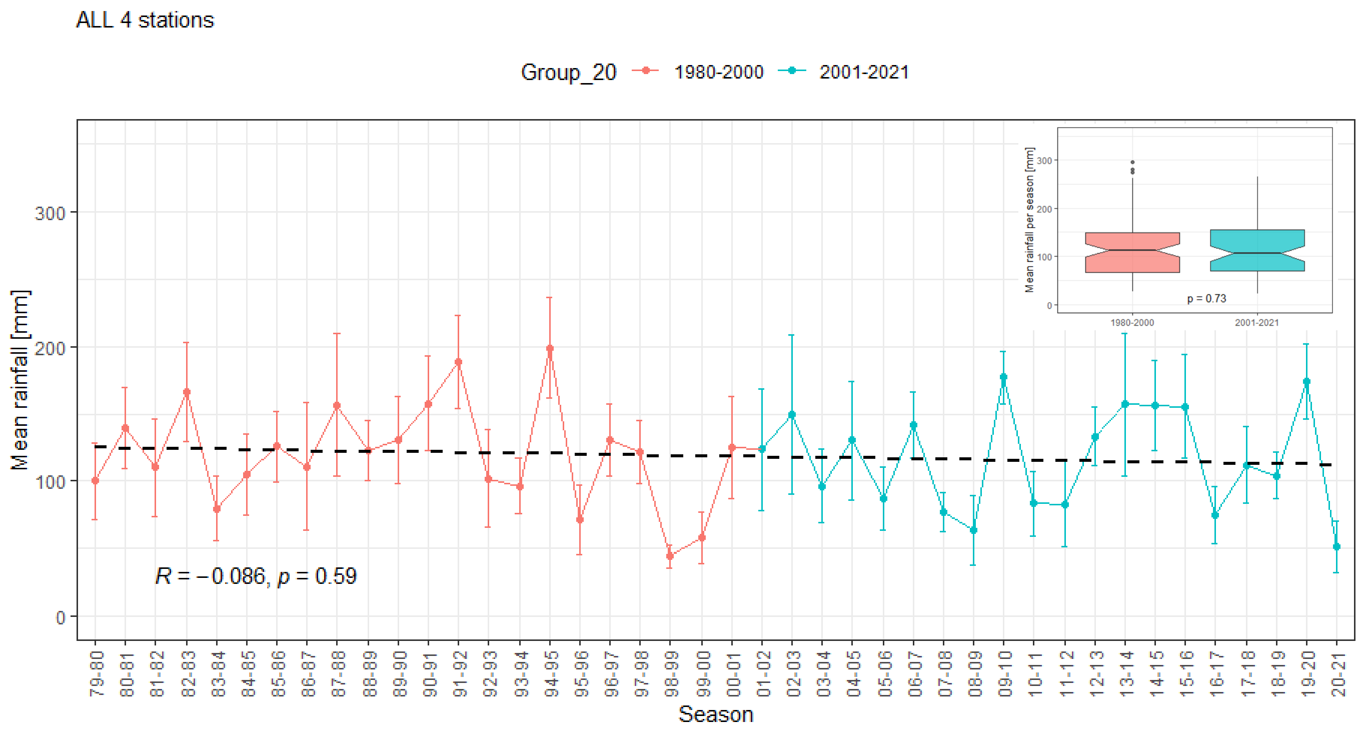

Figure S1. Mean annual rain from 1979 to 2021. There is a slightly but not significant decrease of rain from 1979 to 2021. Boxplots in the corner show the comparison of rainfall by 20-year lapse, there is no significant difference. Season includes from October to May of the next year. Dots represent means ± SE based on the four stations. Slightly decrease but not significant decrease was observed in the last 40 years (dashed line).

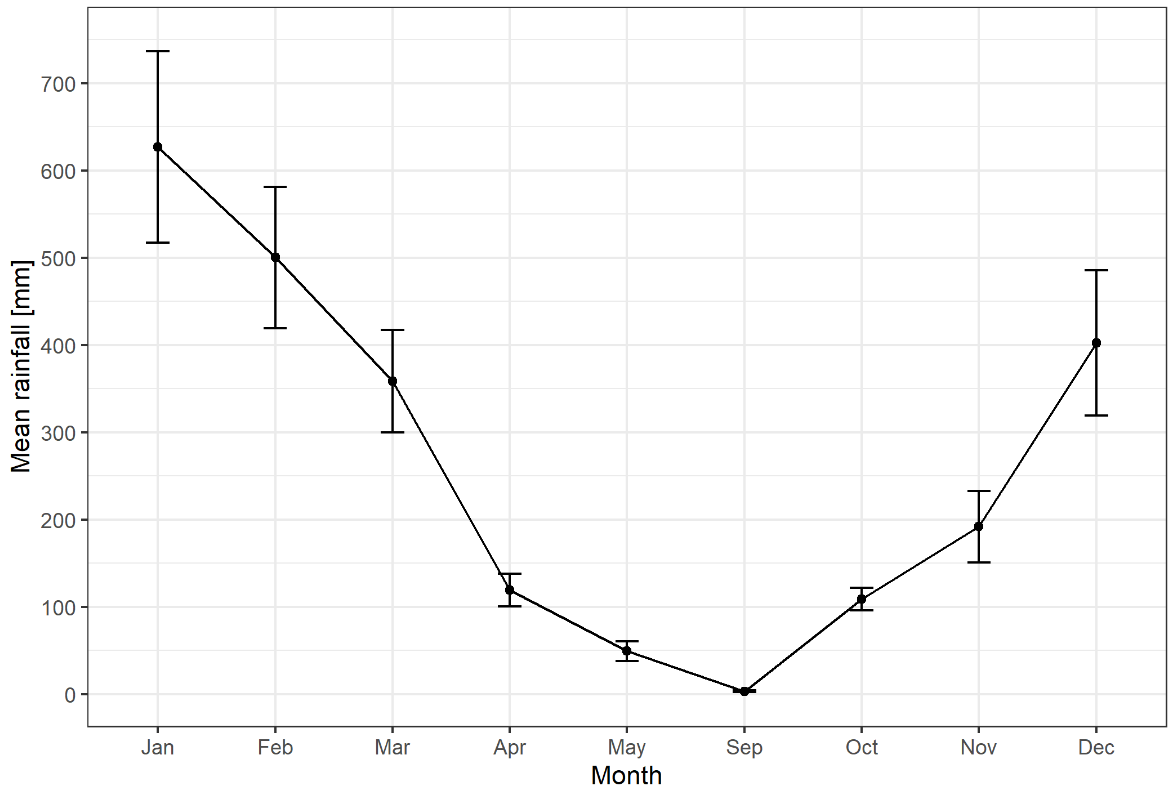

Figure S2. Mean annual rain per month. Rainy season starts in September until May with the highest values in December, January and February. From June to August there is no rain (months not shown, rain = 0). Dots represent means ± SE based on the four stations.

Table S1. Summary of rainfall annual, flower size, black patch area and percentage of black patch area. SD=Standard Deviation, SE= Standard Error

Year	Sample size	Mean	SD	SE	Variable
**1998**	4	121.45	46.63	23.31	Rainfall [mm]
**1999**	4	44.50	16.75	8.37	
**2017**	4	74.95	41.35	20.68	
**2019**	4	104.03	34.41	17.20	
**2020**	4	174.13	56.38	28.19	
**2021**	4	51.20	38.13	19.07	
**1998**	30	44.33	10.41	1.90	Flower size [cm^2]
**1999**	16	37.20	7.03	1.76	
**2017**	289	17.13	5.04	0.30	
**2019**	366	33.71	8.05	0.42	
**2020**	214	40.01	8.73	0.60	
**2021**	33	18.57	5.76	1.00	
**1998**	30	1.05	0.36	0.07	Patch area [cm^2]
**1999**	16	1.17	0.43	0.11	
**2017**	289	1.02	0.42	0.02	
**2019**	366	1.25	0.51	0.03	
**2020**	214	0.98	0.41	0.03	
**2021**	33	0.62	0.28	0.05	
**1998**	30	2.46	0.87	0.16	Percentage of patch area [%]
**1999**	16	3.13	0.96	0.24	
**2017**	289	6.07	2.59	0.15	
**2019**	366	3.84	1.56	0.08	
**2020**	214	2.43	1.02	0.07	
**2021**	33	3.29	1.28	0.22	
**1998**	1	20.4	---	---	Temperature [°C]
**1999**	1	19.6	---	---	
**2017**	3	19.5	0.73	0.42	
**2019**	4	18.3	0.62	0.33	
**2020**	4	19.5	0.96	0.48	
**2021**	4	19.9	0.93	0.46	

Table S2. ANCOVA. Coefficients and ANOVA results of model: Black patch area ~ rainfall * flower size.

	Estimate	Std. Error	*Df*	Sum Sq	F value	P value
**Flower size [cm^2]**	0.038	0.004	1	13.72	68.69	< 0.001***
**Annual rainfall [mm]**	0.004	0.001	1	6.55	32.79	< 0.001***
**Flower size * Annual rainfall**	-0.002	0.00004	1	3.88	19.42	< 0.001***
**Residuals**			742	148.24		
